# Fermionic wave functions from neural-network constrained hidden states

**DOI:** 10.1073/pnas.2122059119

**Published:** 2022-08-03

**Authors:** Javier Robledo Moreno, Giuseppe Carleo, Antoine Georges, James Stokes

**Affiliations:** ^a^Center for Computational Quantum Physics, Flatiron Institute, New York, NY 10010;; ^b^Center for Quantum Phenomena, Department of Physics, New York University, New York, NY 10003;; ^c^Institute of Physics, École Polytechnique Fédérale de Lausanne, CH-1015 Lausanne, Switzerland;; ^d^Center for Quantum Science and Engineering, Ecole Polytechnique Fédérale de Lausanne (EPFL), CH-1015 Lausanne, Switzerland;; ^e^Collège de France, 75005 Paris, France;; ^f^Centre de Physique Théorique, Ecole Polytechnique, CNRS, 91128 Palaiseau Cedex, France;; ^g^Department of Quantum Matter Physics, University of Geneva, 1211 Geneva 4, Switzerland;; ^h^Center for Computational Mathematics, Flatiron Institute, New York, NY 10010

**Keywords:** quantum physics, neural networks, variational Monte Carlo, electronic structure, fermions

## Abstract

We introduce a systematically improvable family of variational wave functions for the simulation of strongly correlated fermionic systems. This family consists of Slater determinants in an augmented Hilbert space involving “hidden” additional fermionic degrees of freedom. These determinants are projected onto the physical Hilbert space through a constraint that is optimized, together with the single-particle orbitals, using a neural network parameterization. This construction draws inspiration from the success of hidden-particle representations but overcomes the limitations associated with the mean-field treatment of the constraint often used in this context. Our construction provides an extremely expressive family of wave functions, which is proved to be universal. We apply this construction to the ground-state properties of the Hubbard model on the square lattice, achieving levels of accuracy that are competitive with those of state-of-the-art variational methods.

## Significance

Large systems of interacting quantum particles present a notorious computational challenge, since they require to solve an eigenvalue problem in an exponentially large dimensional space. The problem can be approached variationally: A trial wave function is proposed, depending on a set of parameters that are determined by an optimization procedure. Neural networks are a great candidate for the task as they provide an extremely flexible family of trial states. We introduce a neural-network-based trial wave function formalism for the study of systems of interacting fermions. This formalism is based on the addition of extra hidden fermions that, aided by neural networks, “mediate” the correlations between the particles in the wave function when projected back on the physical space.

Many-body quantum systems are computationally challenging because of the exponential dependence of the size of the Hilbert space on the number of particles. Variational approaches address this problem by considering a class of wave functions depending on a set of parameters over which an optimization is performed. In this way, the computationally intractable search over the full Hilbert space is reduced to a search over a submanifold of dimension merely polynomial in the number of particles. Variational approaches have proved successful in providing qualitative and quantitative insights into the nature of the ground state and the low-energy excited states of a number of interacting quantum systems. For example, in the case of spin systems with arbitrary pairwise interactions, it has been proved (1, 2) that the ratio between the energy of optimized mean-field states and the true ground-state energy approaches a finite constant in the limit of large system size. The subsequent development of systematically improvable variational wave functions has led to quantitative agreement with exact energies of one-dimensional systems using matrix product states and recently also in two dimensions using neural-network and tensor-network states (3, 4).

The remarkable success of variational states in the description of quantum spin systems unfortunately does not have a parallel in correlated systems of fermions, however. It is known, for example, that the natural mean-field analog of direct-product states, the so-called Slater determinant (SD) states, fails to even qualitatively describe the thermodynamic limit of Fermi–Hubbard-type Hamiltonians (2) and the development of systematically improvable neural-network–based trial wave functions is currently an active field of research both in second quantization (5–7) and in first quantization (8–14). In the latter approach, the wave-function amplitudes must be antisymmetric functions of the particle configurations, while being able to capture correlations beyond the single-particle Slater determinants. This is typically achieved either by considering determinants of multiparticle orbitals (9, 11, 13) (backflow transformations) or by Slater determinants of single-particle orbitals multiplied by a neural-network Jastrow factor that depends on the lattice occupations (8, 10). Despite being universal in the lattice, the Slater neural-network Jastrow wave functions seem to struggle to get competitive energies in the strong-coupling regime.

The Hubbard model on the square lattice has been the subject of intense theoretical scrutiny and constitutes the most iconic “simple” model of an interacting quantum system. Despite this simplicity, a full computational solution is still to be achieved. For this model, as well as related lattice models of interacting fermions such as the *t*-*J* and Kondo lattice models, significant insight has been obtained using hidden-particle approaches.* Although a number of different formulations are available (15–33), all such approaches share a basic concept that consists of augmenting the physical Hilbert space by auxiliary degrees of freedom and subsequently performing a projection back to the subspace of physical states. This projection can be regarded as a constraint that selects the representative states in the augmented space that are identified with the basis of the physical Hilbert space. In many cases, a mean-field saddle-point approximation is applied both to the auxiliary particle Hamiltonian and to the treatment of the constraint, which is implemented with static and uniform Lagrange multipliers. This mean-field approximation is uncontrolled in general, except when the saddle point is associated with the limit of large number of flavors (17, 19). Even in those cases, going beyond the saddle-point level is challenging and no systematic improvements beyond the mean-field variational wave functions are available, especially in view of the approximate treatment of the constraint.

In this article, we draw inspiration from hidden-particle approaches to construct a systematically improvable family of variational fermionic wave functions. These states are obtained as the exact projection of Slater determinant states in a Hilbert space augmented by hidden-fermionic degrees of freedom. One of the major novelties of the proposed method is that the constraint is parameterized by neural networks, giving rise to an extremely flexible family of wave-function ansätze. The constraint is optimized together with the orbitals in the enlarged Hilbert space with the goal of minimizing the energy. The expressive power of this class of wave functions is demonstrated in a variational Monte Carlo (VMC) setting, obtaining an accuracy that is competitive with the state of the art for the ground-state properties of the Hubbard model in the square and rectangular lattices.

This paper is structured as follows: We begin (*Section 1*) by introducing the Hamiltonian and the physical degrees of freedom of the problem. In *Section 2*, we introduce the fundamentals of the hidden-fermion representation, describe the Slater determinant in the augmented space together with the fully parameterized constraint function, and prove the universality of this representation. *Section 2* also contains details on the VMC implementation. In *Section 3*, we present ground-state energy benchmarks for the Hubbard model with increasingly large system sizes and demonstrate that we can stabilize competing orders of charge and spin stripes for the Hubbard model on rectangular lattice geometries.

## 1. Background: States and Hamiltonian

In this paper we develop a general technique for approximating the ground state of interacting fermionic Hamiltonians with discrete degrees of freedom—as defined for example by discrete orbitals or spatial coordinates. As a specific application, we focus here on the Fermi–Hubbard model, whose Hamiltonian reads

[1] $$\hat{H}=-\sum_{\sigma \in \{↑,↓\}}\sum_{\{i,j\}\in E}t_{ij}({\hat{c}}_{i\sigma }^{†}{\hat{c}}_{j\sigma }+{\hat{c}}_{j\sigma }^{†}{\hat{c}}_{i\sigma })+\sum_{i\in V}U_{i}{\hat{n}}_{i↑}{\hat{n}}_{i↓},$$

where the binary index σ∈{↑,↓} labels two species of fermionic modes satisfying the canonical anticommutation relations,

[2] $$\{{\hat{c}}_{i\sigma }^{†},{\hat{c}}_{j\sigma \prime }\}=\delta_{ij}\delta_{\sigma \sigma \prime },\;\;\{{\hat{c}}_{i\sigma },{\hat{c}}_{j\sigma \prime }\}=0.$$

The fermionic modes ${\hat{c}}_{i\sigma }$ are the physical (electronic) degrees of freedom (DOF). The fermion dynamics are described by the lattice with V sites defined by the nonzero entries of the *t_ij_* hopping matrix, as well as by the onsite coulomb repulsion *U_i_*. In the following, we exclusively focus on the square and rectangular lattices with uniform hopping ($t_{ij}=1$) and onsite repulsion, leaving more general geometries to future studies.

In this work we are concerned with the subspace of definite particle numbers $N_{↑}$ and $N_{↓}$ of the individual spin species, in which case the two species are distinguishable from each other. However, it is convenient to impose full antisymmetry between the spin species to enhance the expressivity of the family of trial wave functions, like in the so-called unrestricted Hartree–Fock (HF). The projection to definite $N_{↑}$ and $N_{↓}$ subspace is imposed in the sampling of the wave-function amplitudes.

## 2. Hidden-Fermion Formalism and Wave-Function Ansatz

### A. States in the Augmented Hilbert Space: Constraint Function.

Recall that the multiparticle physical Hilbert (Fock) space is spanned by M≔2V creation operators ${\hat{c}}_{i\sigma }^{†}$ applied in all possible ways to the Fock vacuum |0〉. The strategy of this paper is to define an augmented Fock space, constructed by $M_{\text{tot}}>M$ fermionic modes.

We partition the mode operators of the augmented Fock space into two species of auxiliary fermionic degrees of freedom ${\hat{a}}_{\mu }^{†}$ and ${\hat{d}}_{\nu }^{†}$, referred to as visible and hidden modes, respectively. We note that, although most hidden-particle approaches enlarge the Hilbert space with bosonic degrees of freedom, fermionic hidden sectors have been considered in recent works (28–33) (see also refs. 22, 23, 34). We require 1≤μ≤M, and $1\leq \nu \leq \tilde{M}$ with $\tilde{M}$ a free hyperparameter. Of course, $M_{\text{tot}}=M+\tilde{M}$. The occupancy of the visible modes ${\hat{a}}_{\mu }^{†}$ is identified one to one with the occupancy of the physical modes ${\hat{c}}_{i\sigma }^{†}$, establishing a direct correspondence between the index *μ* of the visible modes and the position-spin multi-index of the physical modes (i,σ).

Thus, the basis for the augmented Fock space is spanned by the set of states

[3] $$|n,\tilde{n}\rangle =\left(\prod_{i,\sigma }{\left({\hat{a}}_{i\sigma }^{†}\right)}^{n_{i\sigma }}\right)\left(\prod_{\mu =1}^{\tilde{M}}{\left({\hat{d}}_{\mu }^{†}\right)}^{{\tilde{n}}_{\mu }}\right)|0\rangle ,$$

where *n* and $\tilde{n}$ label the occupancy of the visible and hidden modes, respectively. Note that this basis does not have a definite number of hidden fermions, even if the visible occupations are constrained to have definite particle number.

Since the augmented Fock space defines a superset of the physical many-body fermionic states, a collection of “representative” states is chosen to span the basis of the physical Hilbert space within the augmented space, similar to the constraint applied in the hidden rotor, spin, or boson formalism (18, 24–26). This choice produces a basis of the correct dimension, eliminating so-called unphysical states. This constraint is applied by the following procedure: For each visible-fermion occupancy *n* a particular hidden-fermion occupancy $\tilde{n}$ is chosen. The arbitrary choice of the population of the hidden modes can be summarized by a constraint function $F(n)=\tilde{n}$.

In the physical subspace the probability amplitude of the spinful fermion occupancy *n* is given by the overlap between the augmented basis states (Eq. **3**) and a given trial state vector |Ψ〉 of the augmented Fock space, where the hidden occupancy $\tilde{n}$ is controlled by the visible occupancy *n* via the constraint

[4] ψ(n)=〈n,F(n)|Ψ〉.

In this work we consider the search of the optimal constraint function, contrary to previous hidden-particle formulations where the constraint is a fixed physically motivated rule. The resulting wave-function ansatz is thus parameterized by both the choice of the state in the augmented space |Ψ〉 and the constraint function *F*(*n*). Since there exist doubly exponentially many constraint functions, an extremely flexible family of correlated trial wave functions is obtained.

It should be noted that the hidden single-particle orbitals ${\hat{d}}_{\mu }^{†}$ define an abstract space of hidden-particle configurations. While one may be concerned by the nature of this abstract space and the form of the orthogonal set of single-particle orbitals that define its basis, in practice, we work in the basis of particle configurations in the orbitals ${\hat{d}}_{\mu }^{†}$. Consequently, the relevant quantity is the combination of single-particle orbitals in the abstract space.

**Fig. 1.**
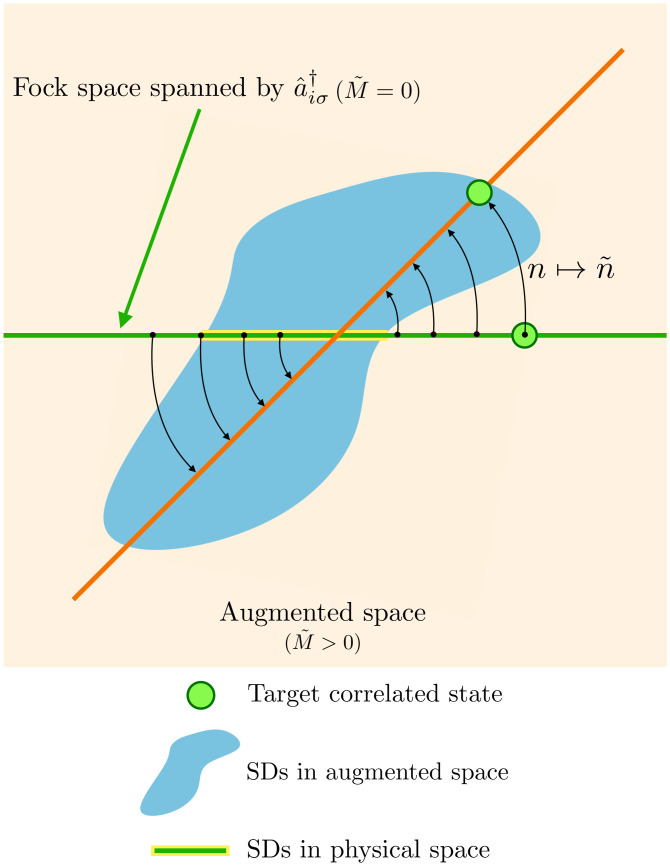
Depiction of the geometrical interpretation of the hidden fermion formalism. The Fock space spanned by the visible-fermionic modes ${\hat{a}}_{i\sigma }^{†}$ is represented by the green horizontal line. The augmented Fock space is represented by the light orange plane (plane of the paper). The orange diagonal line represents the subspace in the augmented Fock space that is isomorphic to the physical Hilbert space after applying the constraint function (black arrows). The collection of SDs in the augmented space is represented by the blue shape, and the intersection with the subspace of just visible DOFs is marked in yellow. This intersection corresponds to the physical Hartree–Fock states. The constraint function changes the collection of states that represent the physical Hilbert space bringing the target correlated state close to a Slater determinant in the enlarged space.

Fig. 1 illustrates geometrically the general concept of the hidden-fermion formalism. The constraint function can be interpreted as a nontrivial rotation of the collection of states that constitute the basis of the physical Fock space embedded in the augmented space (light green horizontal line is rotated to the orange segment in Fig. 1). The goal of this transformation is to bring the target correlated state close to the parameterized family of states in the enlarged space. In Fig. 1 the chosen family of parameterized states is the family of SDs. We also show that in the particular case of $\tilde{M}=0$ (light green subspace) the physical Fock space is directly spanned by the visible modes and that standard SD states can be recovered in that limit.

### B. Hidden-Fermion Determinant States.

#### B.1. Generalities.

To demonstrate the versatility of the hidden-fermion approach, we consider in this work the special case where |Ψ〉 is the uncorrelated Slater determinant state $|\Psi_{\text{SD}}\rangle$, which is characterized by a total number $N_{\text{tot}}\geq N$ of orbital functions $\phi_{n}:\{1,\ldots M_{\text{tot}}\}\to \mathbb{C}$, where $1\leq n\leq N_{\text{tot}}$ and $\tilde{N}=N_{\text{tot}}-N$ is the number of added hidden fermions. In particular, $|\Psi_{\text{SD}}\rangle$ is obtained from the Fock vacuum as

[5] $$|\Psi_{\text{SD}}\rangle ={\hat{\varphi }}_{1}^{†}\cdots {\hat{\varphi }}_{N_{\text{tot}}}^{†}|0\rangle ,$$

where each ${\hat{\varphi }}_{\alpha }^{†}$ is a linear combination of the original creation operators, whose coefficients are determined by the corresponding orbital. In terms of the row vectors

[6] $$({\hat{\varphi }}_{1}^{†},\ldots ,{\hat{\varphi }}_{N_{\text{tot}}}^{†})=({\hat{a}}_{1}^{†},\ldots ,{\hat{a}}_{M}^{†},{\hat{d}}_{1}^{†},\ldots ,{\hat{d}}_{\tilde{M}}^{†})\;\Phi ,$$

where Φ is the $M_{\text{tot}}\times N_{\text{tot}}$ matrix whose columns correspond to the orbital functions. It will be convenient to write the matrix of orbitals in the block form

[7] $$\Phi =\left[\begin{array}{cc} \phi_{\text{v}} & \chi_{\text{v}}\\ \phi_{\text{h}} & \chi_{\text{h}} \end{array}\right],$$

where $\phi_{\text{v}}$ is the *M* × *N* matrix representing the amplitudes of the visible orbitals evaluated in the visible modes, $\chi_{\text{v}}$ is the $M\times \tilde{N}$ matrix representing the amplitude of the hidden orbitals evaluated in the visible modes, $\phi_{\text{h}}$ is the $\tilde{M}\times N$ matrix representing the amplitude of the visible orbitals evaluated in the hidden modes, and $\chi_{\text{h}}$ is the $\tilde{M}\times \tilde{N}$ matrix representing the amplitude of the hidden orbitals evaluated in the hidden modes.

Since the SD state is an eigenstate of the total number operator, as are both the visible and hidden sectors, and anticipating that particle configurations are sampled in the VMC framework, we can represent the constraint as a mapping between the visible-particle configuration $x=(x_{1},\ldots ,x_{N})$ and the hidden-particle configuration $\tilde{x}=({\tilde{x}}_{1},\ldots ,{\tilde{x}}_{N})$:

[8] $$f:x\mapsto \tilde{x}.$$

To respect the Fermi statistics, it is sufficient to choose the function *f* to be of bosonic nature, that is, invariant under permutations of the visible configuration. The amplitudes of the wave-function ansatz in the configuration basis are thus given by

[9] $$\psi (x)=\langle x,f(x)|\Psi_{\text{SD}}\rangle =\det\;\left[\begin{array}{cc} \phi_{\text{v}}(x) & \chi_{\text{v}}(x)\\ \phi_{\text{h}}(f(x)) & \chi_{\text{h}}(f(x)) \end{array}\right],$$

where $\left[\phi_{\text{v}}(x),\;\chi_{\text{v}}(x)\right]$, and $\left[\phi_{\text{h}}(f(x)),\chi_{\text{h}}(f(x))\right]$ denote the $N\times (N+\tilde{N})$ and $\tilde{N}\times (N+\tilde{N})$ submatrices obtained from $\left[\phi_{\text{v}},\chi_{\text{v}}\right]$ and $\left[\phi_{\text{h}},\chi_{\text{h}}\right]$, respectively, by slicing the row entries corresponding to *x* and *f*(*x*). For convenience we denote the $\left[\phi_{\text{h}}(f(x)),\chi_{\text{h}}(f(x))\right]$ matrix as the hidden submatrix.

#### B.2. Universality and connection to other wave-function ansätze.

This ansatz is universal in the lattice. The proof relies on the ability of the determinant in Eq. **9** to represent a universal lookup table of amplitudes that are matched with the amplitudes of an arbitrary target state. In the particular case of $\phi_{\text{h}}=0$ and $\chi_{\text{v}}=0$ the flexibility of the ansatz is relied upon $\chi_{\text{h}}$, as $\phi_{\text{v}}$ leads to amplitudes that correspond to an uncorrelated state in the physical space. It is possible to construct the lookup table for $\tilde{N}\geq 1$, requiring $\tilde{M}$ to grow combinatorially fast with the number of physical fermionic modes. See *SI Appendix* for a detailed discussion. It follows that in the general case where $\phi_{\text{h}}\neq 0$ and $\chi_{\text{v}}\neq 0$, the determinant in the enlarged Fock space does not inherit the nodes of the $\phi_{\text{v}}$ orbitals.

Our construction bears some similarities to backflow transformations (35–38), in which orbitals are taken to be functions of the coordinates of all particles. In contrast to regular backflow, only the restriction of orbitals to hidden states has multiparticle position dependence (Eq. **9**).

Jastrow-like wave-function ansätze of the form

[10] $$\psi_{\text{J}}(x)=J(n)\det\;[\phi_{\text{v}}(x)],$$

where *J*(*n*) is an arbitrary function of the visible lattice occupations, also appear naturally in this formalism. This connection clearly materializes by considering $\tilde{N}=1$ and $\chi_{\text{v}}=\phi_{\text{h}}=0$. In this case the amplitudes of the wave-function ansatz are the product of $\det\;[\phi_{\text{v}}(x)]$ and a symmetric function of the visible-particle configuration $\chi_{\text{h}}(f(x))$. Note that this class of wave functions includes the physically motivated Gutzwiller and Jastrow factors, as well as generalized neural-network Jastrow factors (8, 10) applied to Slater determinants. The constraint function reproducing the Gutzwiller state can be found in *SI Appendix*.

Configuration-interaction (CI) wave functions are also explicitly connected to the hidden-fermion determinant state. Using the Laplace expansion of the determinant in Eq. **9** along its last $\tilde{N}$ rows yields a linear combination of *N*-particle Slater determinants. If $\phi_{\text{V}}$ and $\chi_{\text{V}}$ are chosen to be the *N* lowest HF orbitals and the first $\tilde{N}$ virtual orbitals, respectively, then a CI wave function is obtained, containing all possible (single to $\tilde{N}$-tuple) excitations to the first $\tilde{N}$ virtual orbitals. See *SI Appendix* for the detailed derivation.

#### B.3. Parameterized constraint function, practical implementation.

In contrast to physically motivated constraint functions, a more general approach involves considering *f*(*x*) to belong to a parameterized family, whose parameters are optimized, together with the orbitals, in the energy minimization. The variational Monte Carlo seeks optimal parameters *θ* for a variational family of wave functions $\psi_{\theta }(x)$, which are assumed to be differentiable with respect to *θ*. Although the requirement of differentiability appears to be in tension with the combinatorial nature of *f*(*x*), this obstacle is easily overcome by parameterizing instead the composition of functions ϕ(f(x)) and χ(f(x)), which appears in the hidden submatrix of the enlarged determinant (Eq. **9**). This parameterization is connected to the notion of a continuous set of orthogonal hidden modes ${\hat{d}}_{\mu }^{†}$, which accounts to $\tilde{M}\to \infty$. Remarkably, this automatically satisfies the condition that $\tilde{M}$ must grow combinatorially with *M* for the determinant in Eq. **9** to be a universal lookup table of amplitudes. The hidden-fermion configurations *f*(*x*) are thus represented by some internal state of the parametric function. However, in practice we are never interested in such an internal state.

**Fig. 2.**
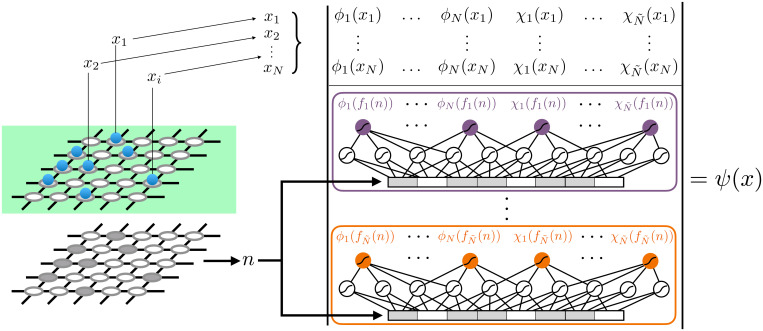
Hidden-fermion determinant-state amplitudes with a neural-network parameterized constraint function. The top part of the determinant is constructed by slicing *N* rows from the top *M* rows of the Φ matrix, according to visible-particle configuration *x*. Each row of the bottom submatrix $\left[\phi_{\text{h}}(f(x)),\chi_{\text{h}}(f(x))\right]$ (hidden submatrix) is parameterized by the outputs of a separate neural network (indicated by different colors), whose input is the flattened visible-lattice occupancy *n*.

Since the hidden submatrix is a matrix-valued function that by construction is a permutation-invariant function of the visible configuration *x*, we choose to represent it by neural networks, taking as an input the visible occupation numbers *n*, without loss of generality. Neural networks are the perfect candidate to reduce the intractable complexity of choosing the optimal constraint, as they define an extremely flexible family of functions. Furthermore, sufficiently large neural networks can represent arbitrary constraint functions, since they satisfy a universal approximation theorem (39). The set of variational parameters *θ* of our ansatz consists of the matrices $\phi_{\text{v}}$ and $\chi_{\text{v}}$ together with the weights and biases parameterizing the corresponding neural network. Fig. 2 details the precise parameterization used in this work. In practice, each row of the hidden submatrix is parameterized by its own neural network, as shown in Fig. 2 by different-colored neural-network blocks. We consider multilayer perceptrons with hyperbolic-tangent activations. The hyperparameters of the ansatz include the neural-network architecture as well as the number of added hidden fermions $\tilde{N}$.

The cost of evaluating the enlarged determinant and its derivatives with respect to the variational parameters scales with the number of visible and hidden fermions as $O({\left(N+\tilde{N}\right)}^{3})$, coming from the used LU (lower-upper) factorization. Typically we choose $\tilde{N}∼O(N)$, and therefore the asymptotic cost of the evaluation of the hidden-fermion determinant state is $O(N^{3})$. The computation of the wave-function amplitudes and gradients is the only step in the VMC algorithm where the required resources are larger, by a constant factor, than those for the computation of an *N*-fermion determinant.

### C. Methods.

Both the amplitudes of the matrices $\phi_{\text{v}}$ and $\chi_{\text{v}}$, together with the weights and biases of the neural networks parameterizing the rows of the hidden submatrix, are jointly optimized using the stochastic reconfiguration method (40), an extension of the classical natural gradient optimization method (41) to variational quantum states. Given that we are interested in the approximation of the ground-state wave function, we rely on the variational principle and use the expectation value of the Hamiltonian with respect to the variational state as the objective function to be optimized. For every Hamiltonian parameter choice a new trial state is optimized from scratch.

General expectation values and gradients of the objective function are computed using Markov chain Monte Carlo sampling according to the probability distribution defined by the square of the wave-function amplitudes $|\psi_{\theta }(x){|}^{2}$, working in the basis of particle configurations. We use the Python library NetKet (42) for the implementation (see *SI Appendix* for details), where gradients of the wave-function amplitudes with respect to the variational parameters are computed by the so-called automatic differentiation implemented in the Python library Jax (43).

## 3. Numerical Experiments

In this section we benchmark the hidden-fermion determinant wave-function ansatz with a fully parameterized constraint function. We first study the square lattice at average site occupation n=1/2 and n=5/8. We use the 4 × 4 square lattice as a test bed to study the accuracy (compared to exact diagonalization [ED]) of the proposed ansatz. The accuracy is quantified by the difference between the ED ground-state energy and the variational energy, relative to the ED ground-state energy. We analyze the effect of the neural network complexity and compare against relevant results in the literature. Finally we focus on rectangular geometries of size 4×L, where we consider 1/8 hole doping (n=7/8). Periodic boundary conditions are set in the short side of the rectangle in all cases. We study the case of both open and periodic boundary conditions on the long side. In the former, we compare our energies with density matrix renormalization group (DMRG) results and study the competing stripe orders of the system. In the latter and in the smallest system size (4×4) we analyze the relative error in the ground-state energy, obtained from ED. In the larger sizes (*L* = 8 and *L* = 16) we compare the ground-state energy with the results obtained using a Slater–Jastrow ansatz and the neural network backflow wave function from ref. 9. In all cases we focus on the zero magnetization and fixed visible- and hidden-particle subspaces.

### A. Benchmarks in the Square Lattice.

We begin by considering the particular case of $\tilde{N}=N$, which provides a good trade-off between computational complexity and accuracy, and a single-hidden-layer neural network parameterizing each row of the hidden submatrix. This architecture is a good starting point to study the effect of the neural-network expressive power in the accuracy of the ansatz. In this case, the expressive power is determined only by the number of hidden units. More hidden units improve the flexibility of the neural network. Furthermore, this single-hidden-layer architecture is the minimal architecture that satisfies the universal approximation theorem (39).

**Fig. 3.**
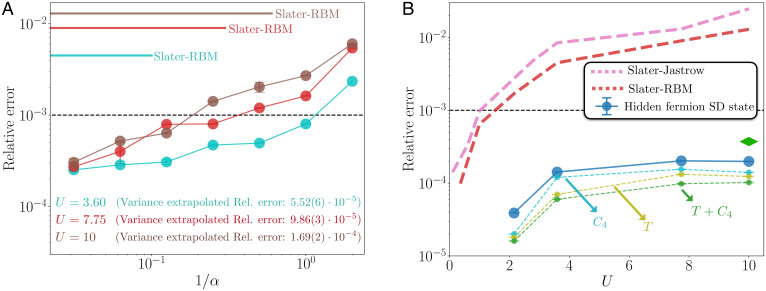
Exact diagonalization benchmarks of the ground-state energy in the 4 × 4 lattice with periodic boundary conditions. (*A*) Relative error in the ground-state energy as a function of the inverse of the width density *α* of the single-hidden-layer neural networks parameterizing the rows of the hidden submatrix. Average physical site occupation is *n* = 1/2 and $\tilde{N}=8$. Different values of *U* are considered, as indicated by each color. The error for a Slater-RBM ansatz (main text) with hidden neuron density *α* = 32, at the same values of *U*, is included for comparison. Indicated is also the relative error from the variance-extrapolated energy for each value of *U* (see *SI Appendix* for details). (*B*) Relative error in the ground-state energy as a function of the coupling constant *U*, at *n* = 5/8 average site occupancy (first closed shell) and $\tilde{N}=10$. The rows of the hidden submatrix are given by single-hidden-layer neural networks with *α* = 64. The errors from Slater–Jastrow and Slater-RBM ansätze are included for comparison. The green diamond is the relative error found with the state-of-the-art, tensor-network–based ansatz from ref. 46. Shown is also the relative error according to the projection of the converged hidden-fermion determinant state to the subspace of invariant wave functions under the action of π/2 rotations (*C*_4_) and the group of all possible translations *T* with *K* = 0 momentum, separately and together.

Fig. 3*A* shows the relative error in the ground-state energy as a function of the ratio between the number of hidden units and input features (*α*), at *n* = 1/2 average site occupation. Different values of *U* are shown, including challenging cases (*U* = 7.75 and *U* = 10) where the ground state is strongly correlated. There is a systematic trend to decrease the error in the energy as *α* is increased, providing a clear and controllable pathway to obtaining more expressive wave-function ansätze. Moreover, for the largest values of *α*, and contrary to what is observed on typical wave-function ansätze, the error does not significantly increase with *U* as the correlations in the ground state increase. Remarkably, the relative error at *U* = 10, typically the most challenging case, is orders of magnitude lower than the error of the Slater–restricted Boltzmann machine (Slater-RBM) wave-function ansatz. The Slater-RBM ansatz is a particular case of the wave function in Eq. **10**, where *J*(*n*) is a restricted Boltzmann machine of complex weights.

The direct extrapolation of the relative error to the α→∞ limit is challenging as the asymptotic scaling of the accuracy with the neural network complexity is not a well-understood matter in the field. However, from the different energy and variance estimates obtained for each *α* we perform an energy-variance extrapolation procedure (44) to obtain better estimates of the ground-state energy. See *SI Appendix* for details. The relative error corresponding to the variance-extrapolated energies is shown in Fig. 3*A*, where the relative error is in this case defined as the difference between the variance extrapolated and the ground-state energies, relative to the ground-state energy.

The improvement of the accuracy with the increase of *α* is accompanied by a gentle increase in the computational complexity of the determinant amplitudes. The scaling with *α* is linear, as the evaluation of the elements of the hidden submatrix requires $O(\tilde{N}(M\cdot \alpha M+\alpha M\cdot (N+\tilde{N})))$ operations, coming from the two affine transformations of the fully connected neural networks with a single hidden layer. For reference, the scaling of the evaluation of the neural-network backflow from ref. 9 is $O(N^{3})$, from the evaluation of the determinant of multiparticle orbitals, while the evaluation of the matrix elements that enter the determinant requires one to store *M* distinct fully connected neural networks and O(N(M·αM+αM·(N)) operations. This makes the asymptotic scaling of the hidden-fermion determinant state with *N*, *M*, and *α* identical to the scaling of the neural-network backflow.

In principle, deeper architectures provide a greater expressive power than their shallower counterparts (45), at the expense of a higher computational cost. We observe that, while deeper architectures provide marginal gains in the energy error, increasing the number of hidden fermions yields a greater impact on the accuracy of the ansatz (see *SI Appendix* for a detailed study of the effect of increasing $\tilde{N}$ and the depth of the neural networks in the accuracy of the ansatz). Benchmarks on physically motivated constraint functions were also performed (see *SI Appendix* for details). Our experiments reveal that parameterizing *f* is advantageous compared to the physically inspired rigid rules, which show a marginal improvement in accuracy compared to the Slater–Jastrow state.

At *n* = 5/8 average site occupation we can compare the relative error in the energy against the state-of-the-art ansatz from ref. 46. *n* = 5/8, which corresponds to *N* = 10, is the first closed shell for the model under consideration in the noninteracting limit. Fig. 3*B* shows the relative error in the ground-state energy as a function of *U*. The error does not increase monotonically with the value of *U*, as standard wave-function ansätze do. This is shown by the reference errors displayed by the Slater–Jastrow and Slater-RBM ansätze. Remarkably, the single Slater determinant ansatz with the parameterized constraint function outperforms (by a factor of 2 in the relative error) the relative error reported in ref. 46 that uses the state-of-the-art ansatz consisting of a pairing reference state multiplied by Jastrow, Gutzwiller, and doublon-holon correlation factors as well as a fat tree tensor network of bond dimension 16, all projected into the zero momentum singlet subspace, with enforced *C*_4_ rotational symmetry. Remarkably, while the result from ref. 46 relies on the projection of the trial state onto given symmetry sectors, our ansatz achieves better accuracy with no symmetry projections. Symmetry projections are an independent avenue to improve the accuracy. So far, we have considered only the increase of the neural-network complexity to obtain better trial states. Not surprisingly, our ansatz is further improved when projected to relevant symmetry subspaces after its convergence, as shown in Fig. 3*B*.

See *SI Appendix* for more benchmarks in square geometries at half filling, where we compare the variational energies from the hidden-fermion determinant state at increasingly larger system sizes with auxiliary field quantum Monte Carlo (AFQMC) calculations (47). Our energies are in better agreement with AFQMC than those obtained with the neural-network Jastrow wave function from ref. 8.

### B. Increasing System Size and Stripe Order at 1/8 Hole-Doped Rectangular Geometries.

To conclude this work, we investigate the validity of the proposed wave-function ansatz on increasingly larger system sizes in rectangular geometries. In particular, we choose rectangular lattices of dimensions L×4, with L={4,8,16}. We focus on the 1/8 hole-doped and zero total magnetization subspace, where, in the strong-coupling regime, the ground state is expected to show hole stripes every eight lattice sites across the long side of the rectangle (λ=8). The high hole density regions coincide with domain walls in the antiferromagnetic order (48, 49). In this section we study the particular case of U=8. For this particular choice of coupling constant and filling, previous works have found different competing orders close in energy to the λ=8 stripe order (48). To guide the wave-function ansatz toward the λ=8 stripe order, we add a soft mean-field constraint to the $\phi_{\text{v}}$ matrix of orbitals. An M×N matrix is added to the variational $\phi_{\text{v}}$. This matrix has zeros everywhere except for a single entry in every column. These entries are filled up with a constant factor S that multiplies $\max(|\phi_{\text{v}}|)$, following the charge and spin order described above. This forces each of the *N* visible orbitals to peak in a certain position of the physical lattice. The value of S is a hyperparameter. The wave function is optimized with this constraint until its convergence, and then the guiding matrix is merged into $\phi_{\text{v}}$ as part of the variational parameters and the energy optimization is continued.

A good trade-off between accuracy and computational resource use is achieved by the addition of $\tilde{N}=16$ hidden fermions for all system sizes. We also consider a two-layer fully connected neural network of hidden-unit density α={60,14,6} for the L={4,8,16} sizes, respectively, to parameterize the rows of the hidden submatrix.

**Fig. 4.**
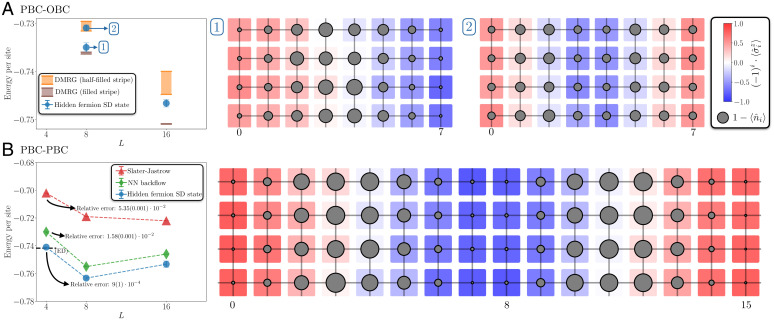
Energy per site and competing charge and spin orders in the 4×L rectangular lattice at 1/8 hole doping (*n* = 0.875) and *U* = 8. (*A*) Periodic boundary conditions on the short side of the cylinder and open on the long side (PBC-OBC). *Left* panel compares the hidden-fermion determinant-state energies with DMRG energies. The width of the DMRG symbols shows the range of converged variational energies for different bond dimensions used in ref. 48. For *L* = 8, blue points labeled as 1 and 2 correspond to filled and half-filled stripes. *Right* panel shows the hole and staggered spin distribution for both metastable configurations. The diameter of the gray circles is proportional to the hole density. (*B*) Periodic boundary conditions along both sides of the rectangles (PBC-PBC). *Left* panel compares the hidden-fermion determinant-state energies with the Slater–Jastrow and neural-network backflow ansätze (from ref. 9). The dashed horizontal line marks the ED (4 × 4 with PBCs from ref. 51) energy. In the 4 × 4 lattice the relative error in the ground-state energy is displayed for each ansatz. *Right* panel shows the hole and staggered spin distributions in the 4 × 16 lattice.

We investigate the case with periodic boundary conditions (PBC) on the short *side* of the rectangle and open boundary conditions (OBC) on the long side (PBC-OBC). Fig. 4 *A*, *Left* shows the energy per site as a function of *L* and the comparison with DMRG variational energies used in ref. 48. The DMRG algorithm finds two metastable solutions, one with half-filled stripes and one with filled stripes. In the *L* = 8 case, we can stabilize both metastable arrangements by tuning the value of S. S=0 or small values of S lead to a half-filled stripe configuration of higher energy. In this system size the charge distribution shows high hole density every four sites, coinciding with domain walls in the antiferromagnetic order. Larger values of S yield a filled-stripe configuration, showing only one stripe of high hole density in the system that coincides with a domain wall in the antiferromagnetic order. The charge and spin configurations for the two competing orders are shown in Fig. 4 *A*, *Right*. They are in good agreement with the DMRG hole distributions. The variational energy from the hidden-fermion determinant state is in good agreement with the DMRG energies. At *L* = 16, the best variational energy found by tuning S lies between the DMRG energies that correspond to the half-filled and filled stripes. Our method, not being specifically tailored to quasi–one-dimensional problems, does not outperform DMRG in this particular lattice geometry.

A more interesting case is the addition of periodic boundary conditions along the long side of the rectangle, a situation that is not amenable to DMRG calculations due to its computational cost. Fig. 4 *B*, *Left* shows the energy per site as a function of *L* and the comparison with the ED energy in the 4 × 4 system. The hidden-fermion determinant with a parameterized constraint function achieves a significantly lower energy than both the standard Slater–Jastrow ansatz and the state-of-the-art neural-network backflow ansatz from ref. 9. In the 4 × 4 lattice, the relative error in the energy is reduced by over one order of magnitude compared to that in the neural-network backflow wave function. In the 4 × 8 and 4 × 16 lattices the energy per site is noticeably lower than that in the neural-network backflow and Slater–Jastrow ansätze. These results demonstrate the scalability of the proposed formalism, which outperforms existing state-of-the-art wave-function ansätze used in the field. For these cases we set S=3.

In addition, we analyze the hole density and staggered spin density distributions in the largest system size (4 × 16) in Fig. 4 *B*, *Right*. The hole density distribution shows repeating maxima separated by eight lattice sites. Coinciding with the maxima in the hole density, the antiferromagnetic order displays a domain wall. The amplitude of the staggered magnetization is modulated along the long side of the rectangles. These features are consistent with observations from previous studies (48, 49) coming from different many-body numerical methods, further validating the accuracy of the hidden-fermion determinant state to find good approximations to the highly correlated ground states of complex Hamiltonians.

## 4. Conclusions

In this paper we have shown that the variational treatment of interacting electrons in an augmented Fock space can be highly beneficial to improve the generality of the wave-function ansatz, especially in the strong correlation limit. We found that key elements for the success of this approach are to optimize the constraint function relating the enlarged and physical Hilbert spaces, as well as treating this constraint exactly. A simple Slater determinant state in the augmented Fock space is found to provide an extremely expressive wave-function ansatz, which we proved to be universal. The optimization of the constraint function and of the hidden-sector orbital amplitudes was performed using a neural-network representation. We presented numerical experiments that show that the proposed wave function is competitive with the state-of-the-art variational accuracy in the ground state of the Hubbard model in the square lattice. Furthermore, in contrast to standard variational approaches (8, 46), the accuracy of this ansatz does not rely on imposing symmetries, potentially allowing for a great level of accuracy on systems with a small number of symmetries. This also opens the possibility of applying our approach to systems without an underlying lattice, with potential applications to quantum chemistry, nuclear physics, and materials science.

In particular, we envision the accurate calculation of the ground-state properties of molecular Hamiltonians, which in the molecular orbital basis lacks both an underlying lattice and exploitable symmetries. The connection between a compact representation of a CI wave function and all possible single-, double-, up to $\tilde{N}$-tuple excitations provides an accurate post-Hartree–Fock starting point for the trial state. The formalism introduced in this paper is also well suited for models with quenched disorder, such as models of interacting fermions on fully connected lattices with random exchange interactions (50). While exact solutions of such models are available in the large-*M* limit when the spin symmetry is extended to *SU*(*M*), the physical *SU*(2) case requires computational approaches. Similar to molecular Hamiltonians, this class of Hamiltonians also lacks any translational symmetries that can be exploited to improve the accuracy of traditional wave-function ansätze.

## Supplementary Material

Supplementary File

## Data Availability

All study data are included in this article and/or *SI Appendix*. Previously published data were used for this work for benchmarking purposes: from ref. 46 (Fig. 3), ref. 48 (Fig. 4*A*), ref. 9 (Fig. 4*B*), and ref. 47 (*SI Appendix*, Fig. S4).

## Footnotes

^*^In the condensed-matter physics literature, the term “slave-particle” representations has been used historically to denote approaches in which the physical Hilbert space is viewed as the projection of an enlarged Hilbert space, often in conjunction with a subsequent mean-field treatment. In this work, we deem it appropriate to use a different terminology and denote by “visible” and “hidden” the previously called “auxiliary” and “slave” degrees of freedom, respectively.
