# Supplementary material for: Fermionic wave functions from neural-network constrained hidden states
Source: Proc Natl Acad Sci U S A. 2022 Aug 3;119(32):e2122059119. doi: 10.1073/pnas.2122059119 (PMC9371695; doi:10.1073/pnas.2122059119)
Supplement: Supplementary File [file pnas.2122059119.sapp.pdf]

1. Universality of the hidden fermion determinant state in the lattice

The aim of this appendix is to prove that the hidden fermion determinant state of amplitudes $\psi(x)$, can exactly match the combinatorially-many amplitudes of an arbitrary target state $\psi_{\mathrm{tar}}(x)$, thus proving $\psi(x)$ to be a universal wave function *ansatz* in the fixed particle subspace of Fock space.

Take N and $\tilde{N}$ visible and hidden fermions occupying M and $\tilde{M}$ visible and hidden modes respectively. For simplicity, and without loss of generality, we consider the particular case where the hidden orbitals have zero amplitude in the visible lattice and the visible orbitals have zero amplitude in the positions of the hidden modes, i.e. $\chi_{\mathrm{v}} = \phi_{\mathrm{h}} = 0$, thus simplifying the evaluation of the amplitudes of the hidden fermion determinant state to:

$$\psi(x) = \det \begin{bmatrix} \phi_{\mathrm{v}}(x) & 0 \\ 0 & \chi_{\mathrm{h}}(f(x)) \end{bmatrix} = \det \left[\phi_{\mathrm{v}}(x)\right] \cdot \det \left[\chi_{\mathrm{h}}(f(x))\right]. \qquad [1]$$

With this particular wave function structure, we already know that $\det\left[\phi_{\mathrm{v}}(x)\right]$ is not capable of matching the amplitudes of an arbitrary state in Fock space. This is because it is a $N \times N$ determinant of single-particle orbitals, thus incapable of capturing correlated states. This implies that the majority of the expressive power of the *ansatz* must come from the $\tilde{N} \times \tilde{N}$ determinant $\det\left[\chi_{\mathrm{h}}(f(x))\right]$. Note that, while the χ_{h} are single particle orbitals of the hidden particle positions, the hidden particle configuration depends on the configuration of all of the visible particles through the constraint function, and consequently, $\det\left[\chi_{\mathrm{h}}(f(x))\right]$ can be interpreted as the determinant of multiple-visible-particle orbitals. Therefore, in order to have $\psi(x) = \psi_{\mathrm{tar}}(x)$, the determinant $\det\left[\chi_{\mathrm{h}}(f(x))\right]$ must satisfy:

$$\det\left[\chi_{\mathrm{h}}(f(x))\right] = \frac{\psi_{\mathrm{tar}}(x)}{\det\left[\phi_{\mathrm{v}}(x)\right]}, \qquad [2]$$

which can only be accomplished iff $\det[\phi_{\mathrm{v}}(x)] \neq 0$ for all x. This requirement is satisfied by choosing the rows of the $(M \times N)$ ϕ_{v} matrix to be non-colinear N-dimensional vectors. Note that both $\psi_{\mathrm{tar}}(x)$ and $\det\left[\phi_{\mathrm{v}}(x)\right]$ are anti-symmetric functions, and in consequence, $\det\left[\chi_{\mathrm{h}}(f(x))\right]$ must be a symmetric function of x, which is achieved by taking $f(x)$ to be symmetric.

Now we provide an explicit construction of $f(x)$ and χ_{h} such that requirement 2 is satisfied for an arbitrary state $\psi_{\mathrm{tar}}(x)$. This construction is based on identifying $\det\left[\chi_{\mathrm{h}}(f(x))\right]$ with a lookup table with $\alpha_{\max} = \binom{M}{N}$ entries, that provides the corresponding amplitudes for the different $\alpha_{\max}$ occupation configurations n of the visible particles on the visible modes. Therefore, in the worst case scenario we require $\tilde{M} = \alpha_{\max} + (\tilde{N} - 1)$ hidden modes. A possible choice for the $\left(\tilde{M} \times \tilde{N}\right)$ matrix of amplitudes χ_{h} that achieves the desired lookup table is given by

$$\chi_{\mathrm{h}} = \begin{bmatrix} \chi_1 & 0 & \dots & 0 \\ \vdots & \vdots & & \vdots \\ \chi_{\alpha_{\max}} & 0 & \dots & 0 \\ 0 & & & \\ \vdots & & \mathbb{I}_{(\tilde{N}-1)} & \\ 0 & & & \end{bmatrix} \qquad [3]$$

with $\mathbb{I}_{(\tilde{N}-1)}$ the $(\tilde{N}-1) \times (\tilde{N}-1)$ identity matrix. We identify $\chi_1, ..., \chi_{\alpha_{\max}}$ with the $\alpha_{\max}$ different amplitudes of the ratio $\psi_{\mathrm{tar}}(x)/\det\left[\phi_{\mathrm{v}}(x)\right]$. The position of hidden fermion i is determined by i^{th} component of the constraint function, i.e. $f_i(x)$, which must satisfy $0 < f_i(x) \leq \tilde{M}$ and $f_i(x) \in \mathbb{Z}$. We choose

$$f_i(x) = \begin{cases} \alpha(x) & \text{if } i = 1 \\ \alpha_{\max} + (i-1) & \text{if } 2 \leq i \leq \tilde{N} \end{cases} \qquad [4]$$

where $0 < \alpha(x) \leq \alpha_{\max}$ and $\alpha \in \mathbb{Z}$ provides a distinct label to each of the $\binom{M}{N}$ different occupation configurations of the visible particles on the visible modes.

With the construction we obtain $\det\left[\chi_{\mathrm{h}}(f(x))\right] = \chi_m = \psi_{\mathrm{tar}}(x)/\det\left[\phi_{\mathrm{v}}(x)\right]$ with $1 \leq m \leq \alpha_{\max}$, thus concluding the proof that the hidden fermion determinant state can match the amplitudes of an arbitrary state in the fixed particle subspace of Fock space.

It is noteworthy that the explicit construction of a combinatorially-large number of hidden modes can be circumvented by directly parametrizing the function composition $\left[\chi_{\mathrm{h}}(f(x))\right]$ by a universal function approximator.

2. Configuration interaction wavefunctions from the hidden fermion determinant state

The aim of this appendix is to provide an explicit construction of a multi-determinant wave function ansatz from the hidden fermion determinant state. In particular, we show that linear combinations of the Hartree-Fock (HF) ground state with all possible single, double, triple,.., $\tilde{N}$-tuple excitations to the $\tilde{N}$ lower virtual orbitals can be constructed from the hidden fermion determinant state with $\tilde{N}$ hidden fermions. First we introduce the notation that will be used in the remaining of the section. Then we show the $\tilde{N} = 1$ and $\tilde{N} = 2$ cases to demonstrate that they lead to a linear combination of N-particle determinants

containing single and double excitations to the first and second virtual orbitals respectively. We conclude by generalizing the result to an arbitrary number of hidden fermions.

$\{\phi_\alpha^{\text{HF}}\}$ is the set of single-particle the HF orbitals. The subscript $\alpha = 1, 2, ..., N_{\text{tot}}$ labels the orbitals in ascending order in energy. We refer to the N lowest energy orbitals as the occupied orbitals. The remaining orbitals are called virtual orbitals, following the quantum chemistry nomenclature. The Slater determinant constructed with $\phi_1^{\text{HF}}, ..., \phi_N^{\text{HF}}$ corresponds to the usual HF ground state $|\Phi\rangle = \hat{\varphi}_1^\dagger \ldots \hat{\varphi}_N^\dagger |0\rangle$, whose amplitudes are given by the determinant:

$$\psi^{\text{HF}}(x) = \det \begin{bmatrix} \phi_1^{\text{HF}}(x_1) & \cdots & \phi_N^{\text{HF}}(x_1) \\ \vdots & & \vdots \\ \phi_1^{\text{HF}}(x_N) & \cdots & \phi_N^{\text{HF}}(x_N)) \end{bmatrix}. \qquad [5]$$

Following the Quantum Chemistry notation $|\Phi_\alpha^\mu\rangle$ with $\alpha \leq N$ and $\mu > N$, is the excitation of the fermion in occupied orbital α to the virtual orbital μ:

$$|\Phi_\alpha^\mu\rangle = \hat{\varphi}_1^\dagger \ldots \hat{\varphi}_{\alpha-1}^\dagger \hat{\varphi}_{\alpha+1}^\dagger \ldots \hat{\varphi}_N^\dagger \cdot \hat{\varphi}_\mu^\dagger |0\rangle. \qquad [6]$$

Analogously $|\Phi_{\alpha_1 \ldots \alpha_n}^{\mu_1 \cdots \mu_n}\rangle$ labels the promotion of the n electrons in occupied orbitals α_1 through α_n to virtual orbitals μ_1 through μ_n.

We choose to identify the visible orbitals ϕ_{v} with the N occupied HF orbitals ϕ_α^{HF} $(1 \leq \alpha \leq N)$ and the $\tilde{N}$ hidden orbitals χ_{h} with the $\tilde{N}$ first virtual orbitalsϕ_μ^{HF} $(N < \mu \leq N + \tilde{N})$.

A. $\tilde{N} = 1$ and single excitations.

According to the above identification of orbitals, the wave function amplitudes become:

$$\psi(x) = \det \left[\begin{array}{cccc} \phi_1^{\text{HF}}(x_1) & \cdots & \phi_N^{\text{HF}}(x_1) & \phi_{N+1}^{\text{HF}}(x_1) \\ \vdots & & \vdots & \vdots \\ \phi_1^{\text{HF}}(x_N) & \cdots & \phi_N^{\text{HF}}(x_N) & \phi_{N+1}^{\text{HF}}(x_{\text{N}})) \\ \hline \phi_1^{\text{HF}}(f_1(x)) & \cdots & \phi_N^{\text{HF}}(f_1(x)) & \phi_{N+1}^{\text{HF}}(f_1(x)) \end{array}\right]. \qquad [7]$$

The cofactor of the above determinant $C_{ij}(x)$ is defined as $C_{ij}(x) = \det[A_{ij}(x)]$, where $A_{ij}(x)$ is the minor of the matrix of orbitals, obtained by eliminating row i and column j from the matrix that enters the original determinant. Using the Laplace expansion of the determinant along its last row:

$$\psi(x) = \sum_{1 \leq \alpha \leq N_{\text{tot}}} (-1)^\alpha \cdot \phi_\alpha^{\text{HF}}(f_1(x)) \cdot C_{N_{\text{tot}},\alpha}(x). \qquad [8]$$

We realize that $C_{N_{\text{tot}},\alpha}(x)$ can be identified with the amplitudes of the N-particle Slater determinant obtained from promoting particle in occupied orbital α to the first virtual orbital. In the particular case where $\alpha = N_{\text{tot}}$, $C_{N_{\text{tot}},N_{\text{tot}}}(x)$ are the amplitudes of the HF ground state. The prefactors to the cofactors can be set to constants $(-1)^\alpha \cdot \phi_\alpha^{\text{HF}}(f_1(x)) = c_\alpha^{N+1}$. For this choice of constraint function and orbital amplitudes, the projected augmented determinant to the physical space corresponds to the CI wavefunction with single-particle excitations:

$$|\psi^{\text{proj}}\rangle = c_0|\Phi\rangle + \sum_{1 \leq \alpha \leq N} c_\alpha^{N+1} |\Phi_\alpha^{N+1}\rangle. \qquad [9]$$

B. $\tilde{N} = 2$ and double excitations.

In this case, the amplitudes of the hidden fermion determinant state are given by the determinant:

$$\psi(x) = \det \left[\begin{array}{ccccc} \phi_1^{\text{HF}}(x_1) & \cdots & \phi_N^{\text{HF}}(x_1) & \phi_{N+1}^{\text{HF}}(x_1) & \phi_{N+2}^{\text{HF}}(x_1) \\ \vdots & & \vdots & \vdots & \vdots \\ \phi_1^{\text{HF}}(x_N) & \cdots & \phi_N^{\text{HF}}(x_N) & \phi_{N+1}^{\text{HF}}(x_{\text{N}})) & \phi_{N+2}^{\text{HF}}(x_{\text{N}})) \\ \hline \phi_1^{\text{HF}}(f_1(x)) & \cdots & \phi_N^{\text{HF}}(f_1(x)) & \phi_{N+1}^{\text{HF}}(f_1(x)) & \phi_{N+2}^{\text{HF}}(f_1(x)) \\ \phi_1^{\text{HF}}(f_2(x)) & \cdots & \phi_N^{\text{HF}}(f_2(x)) & \phi_{N+1}^{\text{HF}}(f_2(x)) & \phi_{N+2}^{\text{HF}}(f_2(x)) \end{array}\right]. \qquad [10]$$

We now proceed to expand the above determinant on its cofactors by its last row, obtaining the linear combinations of $(N+1)$-particle determinants:

$$\psi(x) = \sum_{1 \leq \alpha \leq N_{\text{tot}}} (-1)^\alpha \cdot \phi_\alpha^{\text{HF}}(f_2(x)) \cdot C_{N_{\text{tot}},\alpha}. \qquad [11]$$

$C_{i,j}$ is the cofactor of the $(N+2)$-particle determinant $C_{ij} = \det\left[A_{ij}\right]$ where A_{ij} is the minor obtained by removing row i and column j from the matrix entering the determinant in Eq. 10. Now $C_{N_{\text{tot}},\alpha}$ are $(N+1)$-particle determinants. The cofactors $C_{N_{\text{tot}},\alpha}$ can also be expanded on their cofactors by their last row, obtaining:

$$\psi(x) = \sum_{\substack{1\le\alpha\le N_{\text{tot}}\\ 1\le\beta\le N_{\text{tot}}-1}} (-1)^{\alpha+\beta+1}\cdot\phi_\alpha^{\text{HF}}(f_2(x))\cdot\phi_\beta^{\text{HF}}(f_1(x))\cdot C_{(N_{\text{tot}}-1,\beta)(N_{\text{tot}},\alpha)}. \qquad [12]$$

Here $C_{(kl)(ij)}$ is the cofactor $C_{(kl)(ij)} = \det\left[A_{(kl)(ij)}\right]$ where $A_{(kl)(ij)}$ is the minor obtained by removing row k and column l from the previously defined minor A_{ij}. Now $C_{(N_{\text{tot}}-1,\beta)(N_{\text{tot}},\alpha)}$ are N-particle Slater determinants obtained by promoting the particles in the occupied orbitals α and β to the two lowest virtual orbitals ϕ_{N+1}^{HF} and ϕ_{N+2}^{HF}. It must be noted that the limiting cases $C_{(N_{\text{tot}}-1,N_{\text{tot}}-1)(N_{\text{tot}},N_{\text{tot}})}$, $C_{(N_{\text{tot}}-1,N_{\text{tot}}-1)(N_{\text{tot}},\alpha)}$ and $C_{(N_{\text{tot}}-1,\beta)(N_{\text{tot}},N_{\text{tot}})}$ are the HF ground-state determinant, the excitation of the particle in occupied orbital α to the second virtual orbital and the excitation of the particle in occupied orbital β to the first virtual orbital receptively. As before, setting the prefactors to the cofactors to be the constants:

$$(-1)^{\alpha+\beta+1}\cdot\phi_\alpha^{\text{HF}}(f_2(x))\cdot\phi_\alpha^{\text{HF}}(f_2(x)) := \begin{cases} c_0 & \text{if } \alpha = N_{\text{tot}};\ \beta = N_{\text{tot}}-1 \\ c_\beta^{N+1} & \text{if } \alpha = N_{\text{tot}};\ \beta \le N \\ c_\alpha^{N+2} & \text{if } \alpha \le N;\ \beta = N_{\text{tot}}-1 \\ c_{\alpha,\beta}^{N+2,N+1} & \text{if } \alpha \le N;\ \beta =\le N \end{cases} \qquad [13]$$

the augmented determinant, projected to the physical space corresponds to the the CI wavefunction with single and double-particle excitations:

$$|\psi^{\text{proj}}\rangle = c_0|\Psi\rangle + \underbrace{\sum_{\substack{1\le\alpha\le N\\ N<\mu\le N_{\text{tot}}}} c_\alpha^\mu\cdot|\Phi_\alpha^\mu\rangle}_{\text{singles}} + \underbrace{\sum_{\substack{1\le\alpha_1<\alpha_2\le N\\ N<\mu_1<\mu_2\le N_{\text{tot}}}} c_{\alpha_1,\alpha_2}^{\mu_1,\mu_2}\cdot|\Phi_{\alpha_1,\alpha_2}^{\mu_1,\mu_2}\rangle}_{\text{doubles}}. \qquad [14]$$

C. General $\tilde{N}$ and $\tilde{N}$-tuple excitations.

The generalization of the two cases above to arbitrary $\tilde{N}$ implies that the hidden fermion determinant state of $\tilde{N}$ hidden fermions can be chosen to be a compact representation of a CI wavefunction with all possible single through $\tilde{N}$-tuple particle excitations to the $\tilde{N}$ lowest virtual orbitals:

$$|\psi^{\text{proj}}\rangle = c_0\cdot|\Phi\rangle + \underbrace{\sum_{\substack{1\le\alpha\le N\\ N<\mu\le N_{\text{tot}}}} c_\alpha^\mu|\Phi_\alpha^\mu\rangle}_{\text{singles}} + \underbrace{\sum_{\substack{1\le\alpha_1<\alpha_2\le N\\ N<\mu_1<\mu_2\le N_{\text{tot}}}} c_{\alpha_1,\alpha_2}^{\mu_1,\mu_2}\cdot|\Phi_{\alpha_1,\alpha_2}^{\mu_1,\mu_2}\rangle}_{\text{doubles}} + \ldots + \underbrace{\sum_{\substack{1\le\alpha_1<\ldots<\alpha_{\tilde{N}}\le N\\ N<\mu_1<\ldots<\mu_{\tilde{N}}\le N_{\text{tot}}}}}_{\tilde{N}-\text{tuples}} c_{\alpha_1\ldots\alpha_{\tilde{N}}}^{\mu_1\cdots\mu_{\tilde{N}}}|\Phi_{\alpha_1\ldots\alpha_{\tilde{N}}}^{\mu_1\cdots\mu_{\tilde{N}}}\rangle. \qquad [15]$$

Note that in the above expression the coefficients $c_{\alpha_1\ldots\alpha_n}^{\mu_1\cdots\mu_n}$ are given, up to a sign, by the product

$$\prod_{1\le i,j\le n}\phi_{\alpha_i}(f_j(x)).$$

D. Concluding remarks.

It must be noted that for arbitrary $\tilde{N}$ not all of the coefficients of the above expansion can be independent from each other. This claim is supported by a simple counting argument. There are $\tilde{N}\cdot(\tilde{N}+N)$ of the $\phi_\alpha^{\text{HF}}(f(x))$ coefficients from the bottom hidden sub-matrix. However, in general , the number of free parameters in the above CI expansion is:

$$\sum_{1\le i\le\tilde{N}}\binom{N}{i}\binom{\tilde{N}}{i},$$

which except for $\tilde{N}=1$ is larger than $\tilde{N}\cdot(\tilde{N}+N)$. While this imposes a constraint on the type of CI wavefunctions that can be obtained with this construction, it must be noted that other successful trial states like unitary coupled clusters also suffer from similar constraints.

While the above construction assumed the staring orbitals to be the occupied and virtual orbitals from the HF solution of the given Hamiltonian, a more general case concerns the choice of arbitrary linear combinations of those orbitals. In practice we allow such arbitrary linear combinations, and therefore, the trial state we use in this work contains, as a particular case, the CI wavefunction.

Finally it is important to remark that in this derivation we have set $\phi_\alpha^{\text{HF}}(f(x))$ to be constants. However, in the general case they will be bosonic functions of the visible particle positions, which leads to Jastrow-like factors in the CI expansion, thus leading to a more general wave function.

3. Physically motivated constraint functions

In this appendix we first show an explicit construction of the Gutzwiller wave function *ansatz* from the hidden fermion determinant state. This construction serves as a motivation to showcase the fact that a simple constraint function leads to a correlated wave function. Inspired by this, we present other physically motivated (non-optimizable) constraint functions, and benchmark them in the 4×4 Hubbard model at quarter occupation. We also study the possibility of adding neural-network-based correlation factors to the hidden fermion Slater determinant state.

A. Explicit construction of the Gutzwiller wave function in the Slater determinant hidden fermion state. In order to gain intuition about the nature of the hidden fermion determinant state, consider as a trivial warmup, the molecular orbital wave function, which has the following Fock-space representation corresponding to $N=2$ fermions in $M=4$ modes,

$$\left[c^\dagger_{1\uparrow}c^\dagger_{2\downarrow} + c^\dagger_{2\uparrow}c^\dagger_{1\downarrow} + e^{-g}(c^\dagger_{1\uparrow}c^\dagger_{1\downarrow} + c^\dagger_{2\uparrow}c^\dagger_{2\downarrow})\right]|0\rangle \ . \tag{16}$$

by introducing one hidden fermion ($\tilde{N}=1$) with two hidden states ($\tilde{M}=2$) and using the following disjointly supported orbitals,

$$\phi_{\rm v} = \begin{bmatrix} \phi_1(1\uparrow) & 0 \\ \phi_1(2\uparrow) & 0 \\ 0 & \phi_2(1\downarrow) \\ 0 & \phi_2(2\downarrow) \end{bmatrix} \ , \qquad \chi_{\rm v} = \begin{bmatrix} 0 \\ 0 \\ 0 \\ 0 \end{bmatrix} \ , \tag{17}$$

$$\phi_{\rm h} = \begin{bmatrix} 0 & 0 \\ 0 & 0 \end{bmatrix} \ , \qquad \chi_{\rm h} = \begin{bmatrix} 1 \\ e^{-g} \end{bmatrix} \ . \tag{18}$$

In particular, with the following choice of constraint function

$$f(x) = n_{1\uparrow}n_{1\downarrow} + n_{2\uparrow}n_{2\downarrow} \ , \tag{19}$$

we find the probability amplitudes

$$\psi(x) = \det \begin{bmatrix} \phi_1(i_1) & \phi_2(i_1) & 0 \\ \phi_1(i_2) & \phi_2(i_2) & 0 \\ 0 & 0 & \exp\left[-g\sum_{i=1}^2 n_{i\uparrow}n_{i\downarrow}\right] \end{bmatrix} \tag{20}$$

$$= \exp\left[-g\sum_{i=1}^{2} n_{i\uparrow}n_{i\downarrow}\right] \det \begin{bmatrix} \phi_1(i_1) & \phi_2(i_1) \\ \phi_1(i_2) & \phi_2(i_2) \end{bmatrix} \ , \tag{21}$$

which is of the required form. By similar reasoning, one can construct the Gutzwiller projection of an arbitrary Slater determinant state with $2M$ modes described by orbitals $\phi_{\rm v}$,

$$\psi(x) = \exp\left[-g\sum_{i=1}^{M} n_{i\uparrow}n_{i\downarrow}\right] \det\left[\phi_{\rm v}(x)\right] \ , \tag{22}$$

simply by choosing disjointly supported hidden and visible orbitals $\phi_{\rm h}=0$, $\chi_{\rm v}=0$, $\chi_{\rm h} = \left[1, e^{-g}, \ldots, e^{-Mg}\right]^{\rm T}$ together with the constraint function

$$f(x) = \sum_{i=1}^{M} n_{i\uparrow}n_{i\downarrow} \ . \tag{23}$$

B. Benchmarks with physically motivated constraint functions. Motivated by the ability to reproduce correlated wave function *ansätze* as described above, we propose and test a collection of constraint functions built from the physical intuition about the ground state physics of the Hamiltonian in Eq. (1) in the main text.

B.1. Gutzwiller-inspired constraint function. Take $\tilde{N}=1$ and $\tilde{M}=N/2$, with the constraint function $f(x)=\sum_{i=1}^{M} n_{i\uparrow}n_{i\downarrow}$. The position of the hidden fermion in the hidden modes is determined by the number of doubly occupied sites in the x configuration. The wave function *ansatz* is the Slater determinant in the enlarged Hilbert space of Eq. (5) in the main text. The variational parameters are the coefficients (orbitals) of the non-orthogonal change of single-particle basis in Eq. (6) in the main text. For a particular choice of orbitals this *ansatz* can reproduce the Gutzwiller wave function as described above.

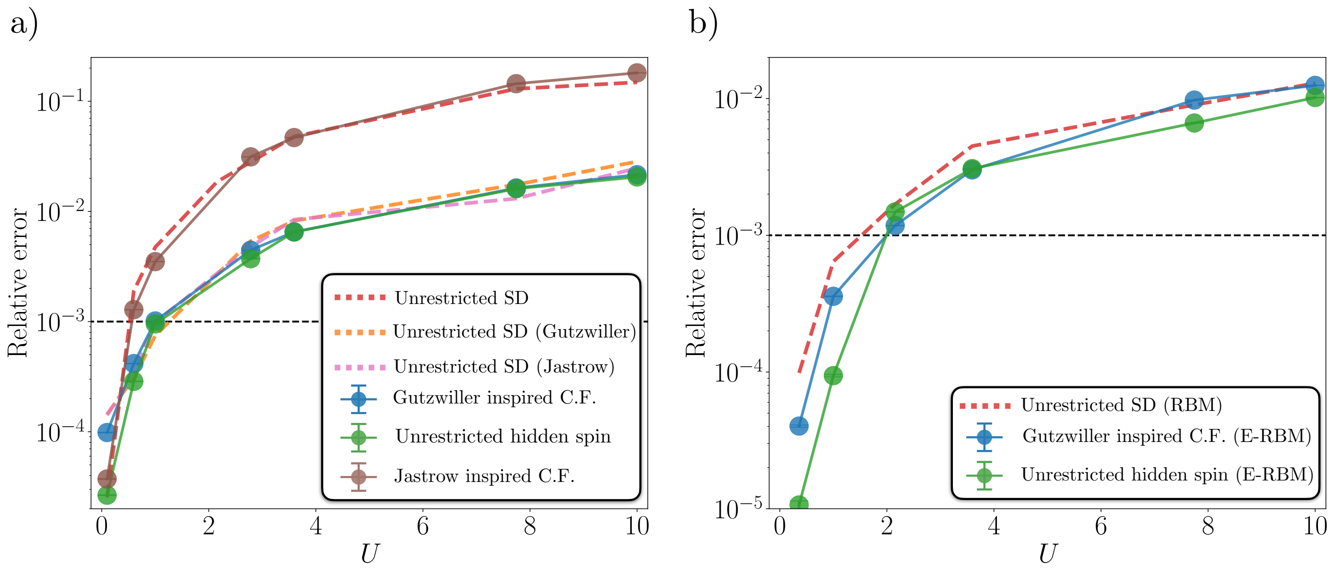

Fig. 1. Benchmarks of physically motivated constraint functions with ED energies in the 4×4 Hubbard model at $n = 1/2$ average physical site occupation. Results from standard wave function *ansätze* are shown as dashed lines for comparison purposes. (a) Relative error in the ground-state energy as a function of the coupling constant U. The different constraint function *ansätze* are a single Slater determinant in the augmented Fock space with no projections. (b) Same as panel (a) including a complex RBM projection factor both in the control unrestricted HF *ansatz* and a E-RBM factor in the the hidden fermion *ansätze*.

B.2. Unrestricted hidden spin ansatz. Consider a pair of hidden fermionic modes $\hat{d}^\dagger_{i\beta}$ with $\beta = 0, 1$, associated to each site of the visible Hubbard lattice i, and a single hidden fermion populating each pair of hidden modes. Thus $\tilde{N} = M/2$ and $\tilde{M} = M$. The position of the hidden fermion depends on the visible site occupancy via the local constraint function $f_i(x) = n_{i\uparrow} n_{i\downarrow} = \beta$. The wave function *ansatz* is the Slater determinant in the enlarged Hilbert space of Eq. (5) in the main text. The variational parameters are the coefficients (orbitals) of the non-orthogonal change of single-particle basis in Eq. (6) in the main text. This *ansatz* is closely related to the hidden spin formalism (1). The difference is to consider the hidden fermions to be indistinguishable particles amongst each other and the visible ones, as opposed to the hidden spin formalism where the added spins are distinguishable particles. This unrestricted hidden spin *ansatz* provides more flexibility, the same way the unrestricted Hartree-Fock does compared to factorised HF. It is easy to see that the unrestricted hidden spin *ansatz* can reproduce the Gutzwiller *ansatz* for a particular choice of orbitals. Furthermore, it was proven that the hidden spin formalism can capture the Mott transition at the mean field level. Therefore, this *ansatz* can capture correlations beyond the Gutzwiller single-site correlations.

B.3. Jastrow-inspired constraint function. Consider a disjointed collection of hidden modes $\hat{d}^\dagger_{l,\beta}$ with $1 \le l \le D_{\max}$ and $0 \le \beta \le N$, where $D_{\max}$ is the maximum graph distance (Manhattan distance) in the physical lattice. There is one hidden fermion for each of the l groups of hidden modes, $\tilde{N} = D_{\max}$. The position of the hidden particle at the lth group of hidden modes depends on the number of visible fermions at distance l in the x configuration:

$$f_l(x) = \left\lfloor \frac{1}{\tilde{N}} \sum_{i \in \mathcal{V}} \sum_{j \in (i+l)} n_i n_j. \right\rfloor \quad [24]$$

B.4. Benchmarks. Panel (a) on Fig. 1 shows the relative error in the ground state energy as a function of U for the Gutzwiller-inspired constraint function, unrestricted hidden spin and Jastrow-inspired constraint function *ansätze*. It also shows the relative error for standard wave function *ansätze* like the unrestricted SD and the unrestricted SD with Gutzwiller and Jastrow factors for comparison. The Gutzwiller-inspired constraint function, and unrestricted hidden spin *ansätze* have a relative error comparable to it of the standard unrestricted SD with Gutzwiller and Jastrow factors, providing for some values of U a marginal improvement in the ground-state energy. On the other hand, the Jastrow-inspired constraint function *ansatz* achieves relative error values comparable to a single unrestricted SD. These results show that the choice of constraint function is of great relevance to find and *ansatz* that can capture correlated phenomena.

In addition to considering mean-field states in the enlarged Hilbert space, correlated states can also be constructed by multiplying the Slater determinant by a parametrized factor that depends on both the occupation of the visible and hidden degrees of freedom. In particular we take that factor to be a complex Restricted Boltzmann Machine in the enlarged space of occupations $n \oplus \tilde{n}$ (E-RBM). Panel (b) of Fig. 1 shows the relative error in the ground-state energy as a function of the coupling constant for the The Gutzwiller-inspired constraint function, and unrestricted hidden spin *ansätze* with an additional E-RBM factor where the hidden layer has twice as many units as input features ($\alpha = 2$). For comparison purposes, we also show the relative error of an unrestricted SD *ansatz* (visible space) with an additional complex RBM factor with twice as many

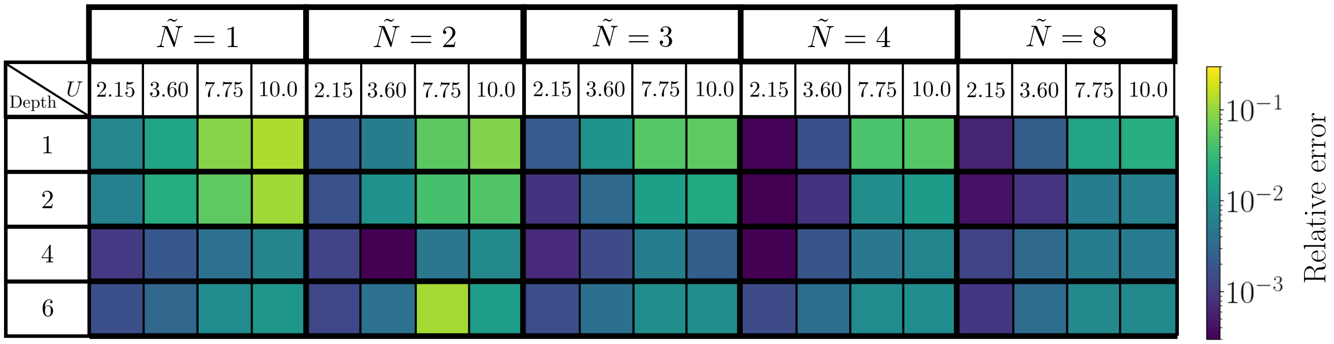

Fig. 2. Effect of the number of hidden fermions and depth of the fully connected neural network that parametrizes the hidden sub-matrix in the expressive power of the hidden fermion determinant $ansatz$. The scale shows the relative error in the ground-state energy for different values of U given N_h and the neural network depth. The results correspond to the 4×4 Hubbard model at $n = 1/2$ filling.

units as input features ($\alpha = 2$), proposed proposed for the first time in Ref. (2). At smaller values of U the correlated *ansätze* in the enlarged space have a lower relative error in the energy than the equivalent correlated *ansatz* in the original Hilbert space. At larger values of U, the correlated unrestricted hidden spin *ansatz* notably outperforms the correlated *ansatz* in the original Hilbert space.

While the results presented above show that the hidden fermion formalism outperforms well established wave functions *ansätze* for carefully chosen constraint functions, it is still unclear how to choose the optimal constraint function. In fact, there exists a doubly exponential number of constraint functions, making the brute-force search of constraint function an intractable problem.

4. Effect of the number of hidden fermions and neural network architecture in the expressive power of the hidden fermion determinant state

We study the effect of the depth of the neural networks, leaving the width of the hidden layers fixed. The width of the first half of hidden layers matches the number of input features and the second half matches the number of output features. Fig. 2 shows the relative error in the ground-state energy for different values of U and $\tilde{N}$ and the neural network depth. For every number of hidden fermions, the accuracy improves as the depth of the neural network increases until a critical value is reached, in this case six hidden layers. Beyond six hidden layers the optimization becomes challenging . Even though deeper architectures are known to be more expressive(3), the optimization of the energy becomes increasingly challenging, making the *ansatz* to get stuck in a local minimum that can be identified with the mean-field solution. It is also noteworthy that for a fixed depth, the error decreases as the number of hidden fermions is increased, to the point that shallow architectures lead to low errors.

In particular, note that a neural network with a single hidden layer (depth 2) and with eight hidden fermions provides the lowest error for the whole range of coupling strengths. We choose this configuration of eight hidden fermions and a single hidden layer architecture as a starting point to study the effect of the width of the network in the *ansatz* expressive power in the main text. The key advantage is that in this network architecture, the number of hidden units is the only control parameter to increase its expressivity. Furthermore, this single hidden layer architecture is the minimal architecture that satisfies the universal approximation theorem(4).

5. Energy-variance extrapolation in Fig. 3 (a)

The convergence of the resulting ground-state energy with the complexity of the neural-networks in the variational states is not a well understood matter, thus, the direct extrapolation of the energy from the parameter controlling the NN complexity is not a well motivated technique. However, it is clear that in general more expressive architectures yield lower energies and variances. This collection of converged energies and variances can be used to extrapolate a better estimate to the ground-state energy.

The energy-variance extrapolation is motivated by the fact that the difference between the true ground-state energy $\langle\hat{H}\rangle_{\mathrm{gs}}$ and the variational expectation value $\langle\hat{H}\rangle$:

$$\delta E = \langle\hat{H}\rangle - \langle\hat{H}\rangle_{\mathrm{gs}} \tag{25}$$

vanishes linearly with the energy variance

$$\Delta E = \frac{\langle\hat{H}^2\rangle - \langle\hat{H}\rangle^2}{\langle\hat{H}\rangle^2} \tag{26}$$

for sufficiently small δE (see Eqs. 2.17-2.23. in Ref (5)). Therefore, a better estimate to $\langle\hat{H}\rangle_{\mathrm{gs}}$ can be extracted by performing linear regression on the variational energies and variances coming from different trial wave function with different NN complexities. In our case for different values of the ratio between hidden and input units α.

The variational energies from Fig. 3 (a) in the main text for different values of α are shown as a function of their variance in Fig. 3, at different values of the coupling constant U. The relation between the variational energy and the variance is in good

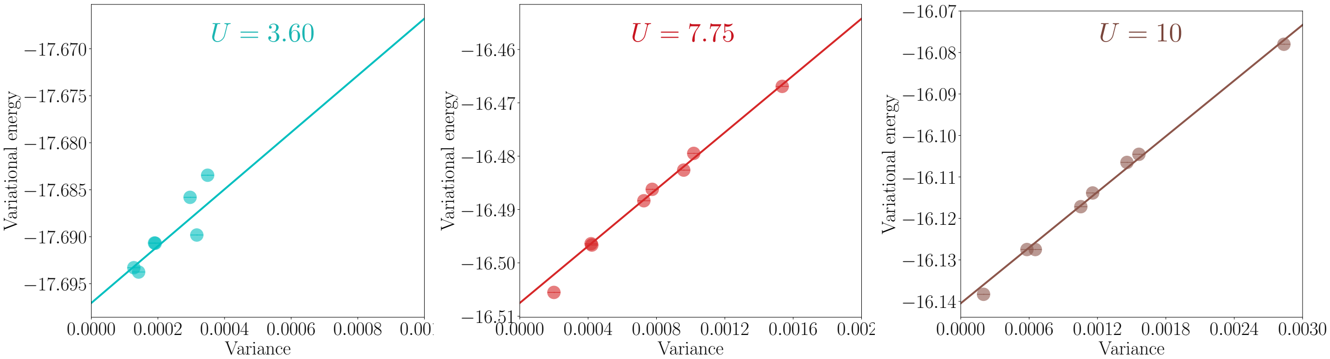

Fig. 3. Energy-variance extrapolation of the ground-state energy for the 4×4 square Hubbard lattice with periodic boundary conditions and average site occupation $n = 1/2$. The variational energy for different values of the ratio between hidden and input units α is shown as a function of its variance. The data is the same as in Fig. 3 (a) in the main text. Different panels show the the extrapolation for different values of the coupling constant U. The solid lines are the lines of best fit to the variational data.

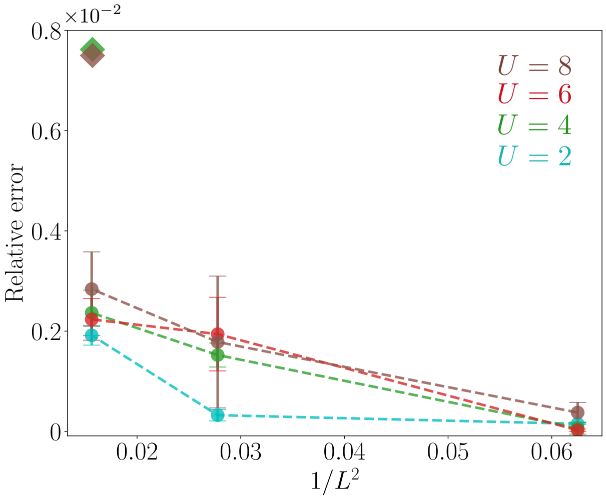

Fig. 4. Relative (to AFQMC energies from Ref. (6)) error in the ground-state energy in the square lattice Hubbard model at half filling for increasing side length L. Periodic boundary conditions are imposed along one of the sides while anti-periodic boundary conditions are imposed along the other side. Different values of U are shown in different colors as indicated. The green and brown diamonds correspond to the accuracy reported in Ref. (2) using a neural-network based Jastrow wave function ansatz for $U = 4$ and $U = 8$ respectively.

agreement with a linear trend. From the intercept of the linear extrapolation with the zero variance axis we extract estimates of $\langle \hat{H} \rangle_{\mathrm{gs}}$. The relative errors of the extrapolated energies are shown in Fig. 3 (a) in the main text.

6. Energy benchmarks in the $L \times L$ Hubbard model at half filling.

We study the accuracy, of the hidden fermion determinant state, relative to Auxiliary Field Quantum Monte Carlo (AFQMC), on increasingly larger systems. At half filling AFQMC does not have a sign problem. Therefore it provides very accurate estimates of the energy values.

We focus on the 4×4, 6×6 and 8×8 system sizes, imposing periodic boundary conditions along one of the sides of the square and anti-periodic along the other side. In this case the energy per site increases monotonically with the side length (6). We use the hidden fermion determinant state with a fully parametrized hidden sub-matrix. The number of hidden fermions and hidden unit densities are $\tilde{N} = \{8, 8, 16\}$ and $\alpha = \{96, 78, 1\}$ for the 4×4, 6×6 and 8×8 lattices respectively. Figure 4 shows the relative error in the ground-state energy as a function of the inverse of the number of sites in the lattice for different values of U. While the error increases with the system size, it always stays in the order $\mathcal{O}\left(10^{-3}\right)$ or lower. Remarkably, the achieved accuracy is better, by at least a factor of two, than the reported accuracy for the neural-network based wave function ansatz in Ref. (2).

7. First quantized fermions on the open-sourced library NetKet: a VMC library for distinguishable particles

Recall that in the canonical quantization of N spinless fermions hopping on an undirected graph $G = (\mathcal{V}, \mathcal{E})$, the configuration space is described by arrays of the form $x = (x_1, \ldots, x_N) \in \mathcal{V}^N$, where we assume some ordering on the vertices, which identifies them with the integers from 1 to $|\mathcal{V}|$. In this section we describe how to reformulate the problem in terms of a local spin model necessary for implementation in the NetKet framekwork. The Hilbert space of the spin model consists of a tensor product of

local Hilbert spaces $\mathbb{C}^{N+1}$

$$\mathcal{H}_{\text{spin}} = \bigotimes_{i \in \mathcal{V}} \mathbb{C}^{N+1} \quad [27]$$

which is spanned by orthonormal basis vectors of the form $|\kappa\rangle$ where $\kappa : \mathcal{V} \to \{0, \dots, N\}$. Recall that each function κ provides lookup table describing the location of a vertex within the configuration $x = (x_1, \dots, x_N)$ (or zero if the vertex is absent). It is clear that $\mathcal{H}_{\text{spin}}$ contains many unphysical states. These unphysical states are avoided in the Markov chain by ensuring a valid initialization and using a transition rule that exchanges the state of two vertices. Now we discuss the implementation of the following operators as local operators on $\mathcal{H}_{\text{spin}}$,

$$n_i \ , \qquad n_i n_j \ , \qquad c_i^\dagger c_j \quad [28]$$

The first is represented by a single-site operator $n : \mathbb{C}^{N+1} \to \mathbb{C}^{N+1}$ given by $n := 1 - |0\rangle\langle 0|$ where 1 denotes the identity operator on $\mathbb{C}^{N+1}$. The second is given by the tensor product operator $n \otimes n$, where the left and right tensor factors represent sites i and j, respectively. Finally, $c_i^\dagger c_j$ is represented by $\big(|0\rangle\langle 0| \otimes n\big)\text{SWAP}$.

8. Converged values of variational energies for different trial wave functions

This section contains the numerical values of the converged ground state energies obtained in this work, with special emphasis on the hidden fermion determinant state (HFDS). We also include energies from other trial states for reference. Exact diagonalization energies used in the benchmarks are also provided for the smaller system sizes.

A. 4×4 Hubbard model with $n = 1/2$.

Here we provide the variational and exact diagonalization (ED) energies for different trial states in the square lattice Hubbard model of size 4×4 and average site occupation $n = 1/2$ and periodic boundary conditions, for various values of U.

Table 1 contains the ground-state variational energies for different values of U obtained with the HFDS wave function with a parametrized constraint function. The hidden sub-matrix is parametrized by single-hidden-layer neural networks of width density α. It also contains the ED energies for reference. The data corresponds to the relative errors shown in Fig. 3 (a) in the main text.

U	ED energy	HFDS ($\alpha = 0.5$)	HFDS ($\alpha = 1$)	HFDS ($\alpha = 2$)	HFDS ($\alpha = 4$)	HFDS ($\alpha = 8$)	HFDS ($\alpha = 16$)	HFDS ($\alpha = 32$)
3.6	-17.6980300(1)	-17.65651(7)	-17.68383(9)	-17.6893(1)	-17.6897(4)	-17.6925(6)	-17.6929(8)	-17.69357(4)
7.75	-16.5091548(2)	-16.4196(4)	-16.4823(6)	-16.4893(3)	-16.4959(4)	-16.4960(3)	-16.502(3)	-16.505(1)
10	-16.1432081(7)	-16.0457(1)	-16.0995(5)	-16.1103(8)	-16.1204(5)	-16.1329(4)	-16.1348(5)	-16.1383(9)

Table 1. Ground-state energy for different values of U in the 4×4 Hubbard model with $n = 1/2$ and periodic boundary conditions. Table contains ED energies for reference. Table also contains variational energies from the hidden fermion determinant state with a parametrized constraint function. The data corresponds to the relative errors shown in Fig. 3 (a) in the main text. The hidden sub-matrix is parametrized by single-hidden-layer neural networks of width density α. See the main text for more details.

Table 2 contains ground-state energies for different variational states, with an emphasis on variational energies from hidden fermion determinant states and hidden fermion determinant states with an added RBM factor in the augmented space with physically motivated constraint functions, as described in Section 3 in the Supplementary information. Other trial states are included for reference. It also contains the exact diagonalization (ED) energies used for benchmarking. The data corresponds to the relative errors shown in Fig. 1 in the Supplementary Information.

B. 4×4 Hubbard model with $n = 5/8$.

Table 3 provides the variational (for the hidden fermion determinant state with a fully parametrized hidden sub-matrix) and exact diagonalization (ED) energies in the square lattice Hubbard model of size 4×4 and average site occupation $n = 5/8$ (first closed shell of the model) and periodic boundary conditions, for various values of U. The hidden sub-matrix is parametrized by single-hidden-layer neural networks of width density α. Projections to different symmetry subspaces are applied to the converged trial state: rotation of $\pi/2$ (C_4), translations of momentum $K = 0$ and the intersection of both subspaces. See the main text for more details. The data corresponds to the relative errors displayed in Fig. 3 (b) in the main text.

C. $4 \times L$ Hubbard model with $n = 7/8$ and U = 8.

Here we present the converged ground-state energies of the hidden fermion determinant state with a fully parametrized hidden sub-matrix in the Hubbard model in rectangular geometries of dimension $4 \times L$ at 1/8 hole doping ($n = 7/8$) and $U = 8$. Periodic boundary conditions are considered along the short side of the rectangle, while both open and periodic boundary conditions are taken along the long side of the rectangle. In Table 4 both configurations are labelled as PBC-PBC and PBC-OBC respectively. Table 4 shows the variational energy per site obtained with the hidden fermion determinant state with a fully parametrized hidden sub-matrix. The value of α depends on the system size (see Section 3. B in the main text for details). The energies are those displayed in Fig. 4 in the main text.

Ansatz	U = 0.1	U = 0.6	U = 1	U =2.15	U = 3.6	U = 7.75	U =10
ED	-19.9073095(9)	-19.4747633(2)	-19.161986(3)	-18.4053208(8)	-17.698030(1)	-16.509154(8)	-16.509154(8)
USD	-19.906(7)	-19.43(3)	-19.07(4)	-18.06(6)	-16.85(8)	-14.36(8)	-13.74(7)
USD (Gutzwiller)	-19.9044(7)	-19.468(1)	-19.147(2)	-18.306(3)	-17.552(1)	-16.219(1)	-15.685(2)
USD (Jastrow)	-19.90449(1)	-19.46832(6)	-19.1432(1)	-18.3173(2)	-17.5487(3)	-16.2932(5)	-15.7443(6)
USD (RBM)	-19.904895(4)	-19.47284(1)	-19.14965(3)	-18.37420(7)	-17.6184(1)	-16.3607(2)	-15.9339(2)
Gutzwiller inspired CF	-19.90535(1)	-19.46667(2)	-19.14257(3)	-18.32346(8)	-17.58269(9)	-16.2395(1)	-15.7953(2)
Unrestricted hidden spin	-19.906781(3)	-19.46918(1)	-19.14371(2)	-18.33683(7)	-17.58288(8)	-15.8113(1)	-16.1697(2)
Jastrow inspired CF	-19.906567(7)	-19.44978(4)	-19.09486(7)	-17.8293(2)	-16.8694(2)	-14.1253(4)	-13.2197(5)
Gutzwiller inspired CF(RBM)	-19.904895(4)	-19.47284(1)	-19.14965(3)	-18.37420(6)	-17.6184(2)	-16.3607(2)	-15.9416(2)
Unrestricted hid. spin(RBM)	-19.904427(5)	-19.47455(1)	-19.16018(3)	-18.37803(5)	-17.64362(9)	-16.3997(2)	-15.9788(2)

Table 2. Ground-state energy for different values of U in the 4×4 Hubbard model with $n = 1/2$ and periodic boundary conditions. Table contains ED energies for reference. The table contains variational energies for various trial states, including unrestricted Slater determinants (USD), unrestricted Slater determinants with applied Gutzwiller factor (USD Gutzwiller), unrestricted Slater determinants with applied Jastrow factor (USD Jastrow), unrestricted Slater determinants with applied RBM factor (USD RBM), as well as HFSD with physically motivated constraint functions: Gutzwiller inspired CF, unrestricted hidden spin and Jastrow inspired CF (see Section 3. B in the Supplementary Information for details). The table also contains results for the HFSD state with an added RBM factor in the augmented space, using the Gutzwiller inspired and unrestricted hidden spin constraint functions (see Section 3. B in the Supplementary Information for details). The data corresponds to the relative errors shown in Fig. 1 in the Supplementary Information.

Ansatz	U =2.15	U = 3.6	U = 7.75	U =10
ED	-21.2122357(7)	-19.8916371(9)	-17.6037315(5)	-16.9035599(8)
HFSD ($\alpha = 64$)	-21.211411(4)	-19.888814(8)	-17.60017(2)	-16.90020(3)
HFSD ($\alpha = 64$)+C_4	-21.211811(4)	-19.889250(7)	-17.60100(2)	-16.90119(2)
HFSD ($\alpha = 64$) + $K = 0$	-21.211853(3)	-19.890244(6)	-17.60140(2)	-16.90147(2)
HFSD ($\alpha = 64$) + ($C_4 \cap K = 0$)	-21.211896(2)	-19.890443(6)	-17.60200(1)	-16.90183(2)

Table 3. Ground state energy for different values of U in the 4×4 Hubbard model with $n = 5/8$ and periodic boundary conditions. Table contains ED energies for reference. The table shows the converged energies for the hidden fermion determinant state with a fully parametrized hidden sub-matrix. Energies of the projection of the converged trial state to different symmetry subspaces are also shown. The data corresponds to the relative errors displayed in Fig. 3 (b) in the main text.

L	Energy per site HFDS (PBC-PBC)	Energy per site HFDS (PBC-OBC)
4	-0.7409(1)	NA
8	-0.7633(7)	-0.7350(6)
16	-0.753(2)	-0.7466(8)

Table 4. Variational energy per site in the Hubbard model in rectangular geometries of dimensions $4 \times L$ at $1/8$ hole doping ($n = 7/8$) and $U = 8$, obtained with the hidden fermion determinant state with a fully parametrized hidden sub-matrix. The value of α depends on the system size (see Section 3. B in the main text for details). The energies are those displayed in Fig. 4 in the main text.

D. $L \times L$ Hubbard model with $n = 1$.

Table 5 shows the energy per site on the square lattice Hubbard model of size $L \times L$ with periodic boundary conditions along one of the sides of the square and anti-periodic boundary conditions along the other side. The trial state is the hidden fermion determinant state with a fully parametrized hidden sub-matrix. Details on the number of hidden fermions and hidden unit densities can be found on Section 6 of the Supplementary Information.

L	U = 2	U = 4	U = 6	U = 8
4	-1.25693(1)	-0.9120(1)	-0.68135(1)	-0.5401(1)
6	-1.2079(1)	-0.8717(2)	-0.6609(4)	-0.5270(3)
8	-1.1900(2)	-0.8621(4)	-0.6574(2)	-0.5244(6)

Table 5. Variational energy per site in the $L \times L$ Hubbard model at half filling with periodic boundary conditions along one of the sides of the square and anti-periodic boundary conditions along the other side. The trial state is the hidden fermion determinant state with a fully parametrized hidden sub-matrix (see Section 6 in the Supplementary Information for more details). The energies correspond to the relative errors shown in Fig 4 in the Supplementary Information.

1. L. de'Medici, A. Georges, and S. Biermann. Orbital-selective mott transition in multiband systems: Slave-spin representation and dynamical mean-field theory. *Phys. Rev. B*, 72:205124, Nov 2005. . URL https://link.aps.org/doi/10.1103/PhysRevB.72.205124.
2. Yusuke Nomura, Andrew S. Darmawan, Youhei Yamaji, and Masatoshi Imada. Restricted boltzmann machine learning for solving strongly correlated quantum systems. *Phys. Rev. B*, 96:205152, Nov 2017. . URL https://link.aps.org/doi/10.1103/PhysRevB.96.205152.
3. Guido Montúfar, Razvan Pascanu, Kyunghyun Cho, and Yoshua Bengio. On the number of linear regions of deep neural networks, 2014.
4. G. Cybenko. Approximation by superpositions of a sigmoidal function. *Mathematics of Control, Signals and Systems*, 2(4):303–314, Dec 1989. ISSN 1435-568X. . URL https://doi.org/10.1007/BF02551274.

5. Tsuyoshi Kashima and Masatoshi Imada. Path-integral renormalization group method for numerical study on ground states of strongly correlated electronic systems. *Journal of the Physical Society of Japan*, 70(8):2287–2299, 2001. . URL https://doi.org/10.1143/JPSJ.70.2287.
6. Mingpu Qin, Hao Shi, and Shiwei Zhang. Benchmark study of the two-dimensional hubbard model with auxiliary-field quantum monte carlo method. *Phys. Rev. B*, 94:085103, Aug 2016. . URL https://link.aps.org/doi/10.1103/PhysRevB.94.085103.
